# “Lucio's Phenomenon” Associated with *Mycobacterium lepromatosis*

**DOI:** 10.4269/ajtmh.15-0439

**Published:** 2016-03-02

**Authors:** Jesús Salvador Velarde-Félix, Gerardo Alvarado-Villa, Lucio Vera-Cabrera

**Affiliations:** Centro de Medicina Genómica, Servicios de Salud de Sinaloa, Sinaloa, México; Unidad Académica Escuela de Biología, Universidad Autónoma de Sinaloa, Sinaloa, México; Centro de Salud Urbano de Guasave, Servicios de Salud de Sinaloa, Guasave, Sinaloa, Mexico; Servicio de Dermatología, Hospital Universitario, Universidad Autónoma de Nuevo León, Monterrey, Mexico

A 49-year-old man born on Sinaloa, Mexico, presented to the consult on December 2014 with ulcerations in the abdominal region and lower extremities with serous fluid, foul smell, and pus, but without lymphadenopathy. Three years before he developed hypopigmented macules in scapular zone, and in November 2014, the first nodules in lower extremities appeared accompanied by fever and pain, as well as erythema nodosum leprosum; the latter was treated with thalidomide for 10 days.

**Figure 1.**
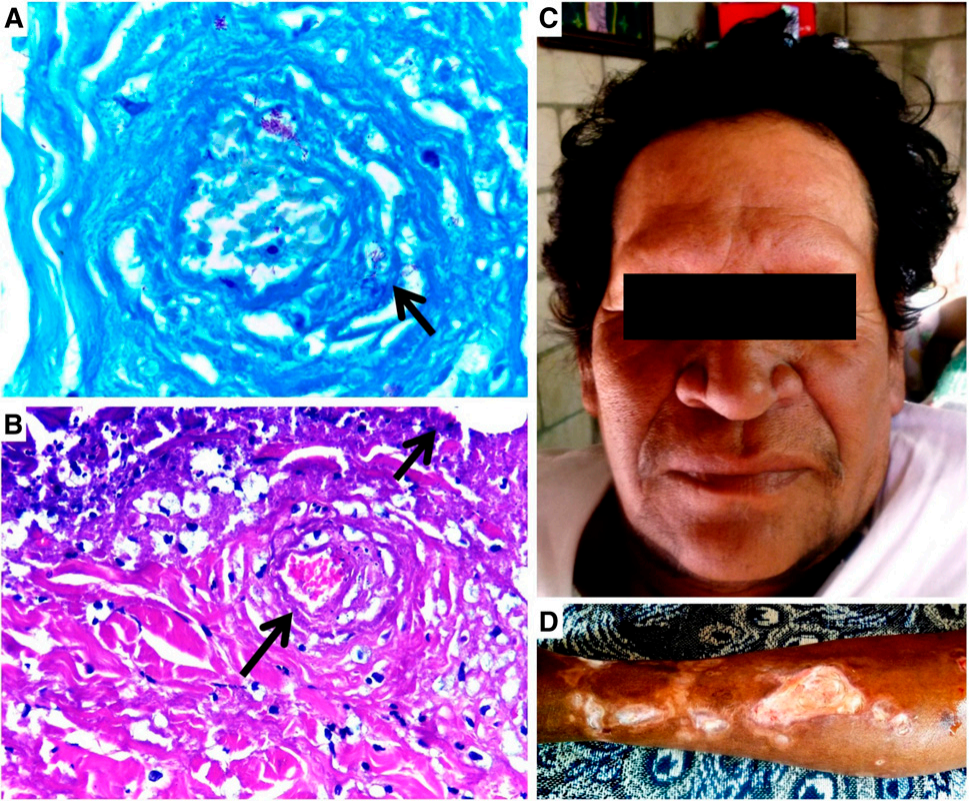
(**A**) Many acid-fast bacilli are seen upon Fite-stained skin (magnification, ×40). (**B**) Fibrinoid necrosis of capillary vessels with onion-skin pattern (lower arrow) and epidermal necrosis (upper arrow) stained with hematoxylin and eosin (Lucio's phenomenon) (magnification, ×10). (**C**) Patient showing diffuse infiltrative, leonine facies with total madarosis (loss of eyebrows, eyelashes). (**D**) Ulcerative dermatitis and erythema nodosum leprosum in left leg.

Bacilloscopy of right earlobe (not shown) and Fite–Faraco stained skin (Figure 1A

) were positive to acid-fast bacilli. Furthermore, chronic granulomatous dermatitis and necrotizing cutaneous vasculitis, characteristic of Lucio's phenomenon (LP) (Figure 1B), were observed.

In February 2015, a fresh tissue biopsy was taken for molecular search of leprosy agents. The results of the followed protocol, described by Vera-Cabrera and others,^1^ allowed us to conclude that only *Mycobacterium lepromatosis* was present in the sample.

In May 2015, the patient started to receive multidrug therapy based on World Health Organization regimen for multibacillary cases. Currently, he presents ulcerative dermatitis (Figure 1D) in abdominal region and lower extremities, leonine facies, both diffuse cutaneous infiltration and loss of sensation in face, complete loss of brows and lashes (Figure 1C), and ulnar neuritis, with grade 2 disability but without neuropathy.

LP is a type III hypersensitivity presented exclusively in diffuse lepromatous leprosy, usually in untreated patients and is characterized by the existence of immune complexes, necrotizing vasculitis on superficial and medium-sized vessels, diffuse infiltration of the skin, dermal necrosis, and sometimes with systemic symptoms. Its prevalence in Mexican patients with lepromatous leprosy has been estimated in 23%, and for many years it was considered endemic in Mexico, but it has also been observed in South America and India.^2^

To date, there are only three reports of LP where *M. lepromatosis* has been identified.^1^,^3^,^4^ The original description and a posterior case using autopsy samples by Han and others,^3^,^4^ and another by Vera-Cabrera and others, who retrospectively described a patient from northeast Mexico.^1^

To the best of our knowledge, the present report constitutes the first LP case associated to *M. lepromatosis* in an alive patient, where one can observe the response to the therapy and possibly study other biological characteristics of *M. lepromatosis* infection.

Written informed consent was obtained from the patient and for all accompanying images. A copy of the written consent was sent to the Editor-in-Chief of this journal.
